# The power of partners: positively engaging networks of people with HIV in testing, treatment and prevention

**DOI:** 10.1002/jia2.25314

**Published:** 2019-07-19

**Authors:** David A Katz, Vincent J Wong, Amy M Medley, Cheryl C Johnson, Peter K Cherutich, Kimberly E Green, Phan Huong, Rachel C Baggaley

**Affiliations:** ^1^ Department of Global Health University of Washington Seattle WA USA; ^2^ Global Health Bureau Office of HIV/AIDS United States Agency for International Development (USAID) Washington DC USA; ^3^ Division of Global HIV and TB Center for Global Health U.S. Centers for Disease Control and Prevention (CDC) Atlanta GA USA; ^4^ HIV Department World Health Organization (WHO) Geneva Switzerland; ^5^ National AIDS/Sexually Transmitted Diseases Control Programme Ministry of Health Nairobi Kenya; ^6^ HIV & TB Program PATH Hanoi Vietnam; ^7^ Vietnam Authority of HIV/AIDS Control, Ministry of Health Hanoi Vietnam

**Keywords:** HIV testing, partner notification, index testing, people with HIV, low and middle income countries, key populations

1

When HIV diagnostic tests first became available in 1985, HIV testing was offered with caution. No treatment was available, prevention options were limited, and stigma and discrimination against people with HIV was pervasive. Concerns about preventing breaches of confidentiality and forced or mandatory testing, especially among groups at high risk for HIV infection, were dominant in early guidance released by the World Health Organization (WHO). An offer of an HIV test required extensive counselling both before and after the test to ensure the individual was able to cope with a possible reactive result. Then the development of point of care rapid HIV tests in 1987 offered the opportunity for immediate results in clinical and community settings 1. When antiretroviral therapy (ART) became available globally in the early 2000s, WHO launched its “3 × 5” Initiative to provide 3 million people in low and middle‐income countries (LMIC) with ART by 2005. Scale‐up of HIV testing through client‐initiated approaches using rapid test kits to provide same day results was a core component to the success of this Initiative 2, 3.

Yet by 2007, HIV testing coverage was still low. Surveys in sub‐Saharan Africa showed that a median of 12% of men and 10% of women had been tested for HIV and received their results 4. In an effort to increase testing coverage, WHO released its Provider‐Initiated Testing and Counselling Guidelines 5, recommending a routine offer of HIV testing by healthcare providers in clinical settings. These guidelines suggest the benefits of HIV status disclosure and partner testing and recommended providers to *discuss possible disclosure of the result, when and how this may happen and to whom* and to *encourage and offer referral for testing and counselling of partners and children* as part of post‐test counselling. However, little emphasis was given to partner testing in practice. Thus, while HIV testing coverage increased enormously over the next five years in clinical settings, especially among pregnant women as part of a push to prevent HIV transmission from mothers to their infants, the vast majority of HIV tests were conducted on people who tested as individuals. Early efforts at partner HIV testing and disclosure were documented, but this approach was rarely implemented and never successfully scaled 6.

Evidence for the benefits of partner testing and disclosure, however, was growing. In addition, population‐based surveys and surveillance efforts indicated that many people with HIV were unaware that they were living in sero‐discordant couples 7, leaving the HIV‐negative partner at high risk for HIV acquisition. This led WHO to recommend partner and couple HIV testing for all adults with HIV in all epidemic contexts and for HIV uninfected individuals living in high HIV burden settings in 2012 8. The preventive benefits of ART 9 and the effectiveness of HIV pre‐exposure prophylaxis (PrEP) 10 in preventing HIV acquisition added to the motivation to scale‐up partner and couple HIV testing.

Over the four years following the release of these guidelines partner testing services were not widely adopted or scaled in most LMICs. However, there were notable exceptions: Cameroon adopted an ambitious partner notification programme in 2007 11; while Kenya, Malawi and Tanzania undertook pivotal implementation studies of partner HIV testing services 12, 13, 14. Rwanda supported male partner involvement and testing at the first antenatal care visit and reported 87% of male partners attending this first visit by 2010 15.

To accelerate uptake of partner testing, WHO released additional guidelines in 2016 on the implementation and scale‐up of ethical, effective, acceptable and evidence‐based approaches to assisted HIV partner notification services (PNS) 16. This guidance was based on a review of available evidence and included the recommendation that voluntary assisted PNS be offered as part of a comprehensive package of testing and care offered to people with HIV. Since its launch, the number of countries including PNS in their national testing policies and guidelines has increased from 77 in 2016 to 111 by the end of 2017 (C Johnson, personal communication), although many countries were not yet implementing PNS at scale.

PNS, or “index testing” as it is referred to in the 2019 President's Emergency Plan for AIDS Relief (PEPFAR) Country Operational Plan Guidance (see A Note on Terminology) are also now prioritized as a major HIV case finding strategy in all 37 PEPFAR‐supported countries and regions. PEPFAR programmes have been actively supporting and expanding index testing approaches since 2016. This expansion has been aided by the development of training materials for index partner and family testing that include information on approaches and steps for conducting PNS, how to screen for intimate partner violence (IPV), brief motivational interviewing as a strategy for partner elicitation, and documentation and monitoring of PNS. PEPFAR also provides technical support to country programmes to assist implementing partners to deliver and monitor PNS 17. In addition, WHO and PEPFAR have supported the development of the AIDSFree HIV Testing Knowledge Base as a publicly available, online resource library of high quality tools (e.g. training materials, standard operating procedures, data collection forms) to support PNS programme development and implementation 18. As a result of this support, implementation of PNS within PEPFAR‐supported programmes has continued to expand. For example, Jhpiego undertook an operational research pilot in Njombe, Tanzania, in 2016 to integrate a free‐choice PNS intervention into health centres which led to successful outcomes at reaching partners 19.

As PNS has expanded, it has also undergone differentiation based on population needs and preferences, ranging from models that centre on assisted PNS provided by healthcare workers, to those that include secondary distribution of HIV self‐testing as part of PNS, to lay providers from key populations using social media and other online tools to trace listed partners. Ongoing challenges to assisted PNS scale‐up include the large amount of human and financial resources needed to support these services, such as the time needed to trace and test partners, particularly if they live outside the jurisdiction of the health facility or community organization. In addition, assisted PNS is not allowed under the laws and policies of many countries. Continued advocacy is needed to modify these policies so that index clients are able to choose the best approach to notifying their partners based on their life circumstances.

Some countries have also been considering or adopting newer HIV testing strategies to increase detection of acute and recent HIV infections. The United States’ *Ending the HIV Epidemic: A Plan for America* suggests that mapping clusters of new HIV infections and offering rapid PNS, ART, and prevention services within identified transmission clusters could disrupt onward transmission 20. As more data and experience from using HIV diagnostics that can detect acute and recent HIV infections in LMICs as part of testing services become available, their acceptability, impact and cost‐effectiveness will guide future recommendations on their role and use.

An indicator for monitoring partner testing cascades has been integrated into PEPFAR's list of required indicators since 2019. This indicator measures both the reach and positivity of index testing offered to the sexual and needle‐sharing partner(s) and biological child(ren) of people with HIV. These data now present partner testing cascades that incorporate the offer of PNS, number of partners and children (i.e. contacts) elicited per index client and downstream uptake and positivity of contacts tested. Examination of these cascades can support improvements to programmes for this new approach by identifying bottlenecks to service delivery. Figure 1 provides an illustration of these partner testing cascades.

**Figure 1:**
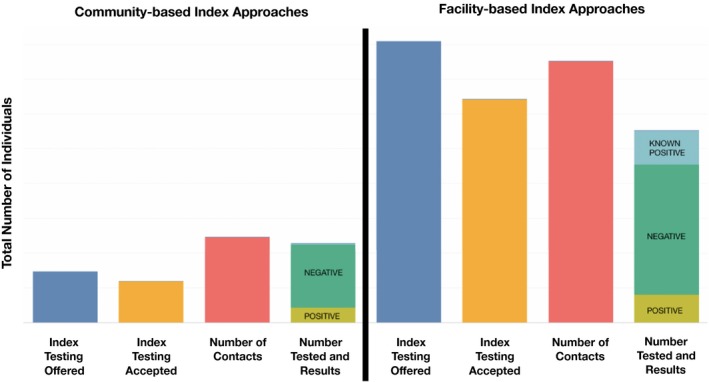
**Illustrative adult partner notification and index testing cascade for community‐ and facility‐based services.**Cascades such as this can be created from data routinely collected by President's Emergency Plan for AIDS Relief programmes. Note that, as currently collected, index client outcomes (index testing offered and accepted) and the total number of contacts are counted where the index client receives services, whereas the number of contacts tested and their results are counted where the contact receives services. As a result, the number of contacts tested and their results may not be counted in the same setting as the index clients who named them, which limits the interpretation of these data as a true “cascade.” For example, index clients receiving services at a facility may indicate partners who are found and tested in the community, and vice versa.

Another important metric to measure is the proportion of partners who report testing as a result of notification by either sex partners or healthcare workers. This will allow programmes to determine the scale and effectiveness of PNS at encouraging partners to seek HIV testing. In Kenya, a PNS surveillance system was created from HIV registries to determine the proportion of individuals who sought HIV testing because they were notified of potential HIV exposure by a sexual partner, by a health provider or some other source. This system allowed the Ministry of Health to determine the coverage of assisted PNS and is now being used to identify populations and areas in need of targeted scale‐up 21.

The articles in this supplement represent a diversity of programmatic experiences, research, and community, provider, and client perspectives on PNS and index testing from 11 countries across sub‐Saharan Africa and Asia. Together, they provide new evidence to support the global focus on PNS as an effective, efficient and safe strategy for achieving the first 90 of the UNAIDS 90‐90‐90 targets, to ensure that 90% of people with HIV are aware of their status 22. The articles also offer practical guidance for implementing PNS in a range of populations and settings and describe new strategies for enhancing PNS delivery. They highlight the need to provide services that meet the WHO minimum standards for HIV testing – consent, confidentiality, counselling, connection to services, and correct test results (the 5C's) 23 – and avoid social harms. Of note, Ayala and colleagues 24 emphasize the importance of involving communities during programme development so that the benefits of PNS are understood and services are provided in an ethical and inclusive way that respects individual choice and human rights.

## 2 Programmatic evidence of assisted PNS effectiveness

PNS are an increasingly prioritized HIV testing strategy globally because of their potential to improve testing coverage and efficiently identify people with undiagnosed HIV infection 16. In this supplement, evaluations of five assisted PNS programmes provide further evidence for the acceptability, sustained effectiveness, and high positivity of new HIV diagnoses among contacts across diverse populations, programme types and settings. HIV positivity rates among contacts (i.e. proportion of contacts tested for HIV who are newly diagnosed as HIV positive) in these programmes ranged from 6% to 15% in Central and South Asia 25, 26 to 29% to 52% in sub‐Saharan Africa 27, 28 and 42% in Southeast Asia 29.

As with any intervention, however, the effectiveness of assisted PNS may depend on the setting or client characteristics, and subgroup analyses have the potential to inform prioritization of PNS when resources are scarce or during programme scale‐up. Masyuko et al. 30 present subgroup analyses of a landmark cluster randomized controlled trial of assisted PNS in Kenya 14, finding that assisted PNS were more effective at increasing partner testing in a region with higher HIV prevalence, in rural and peri‐urban settings with less access to HIV testing services (HTS) and for female index clients. Similarly, an evaluation of a community‐based PNS programme in Zimbabwe found greater test positivity among contacts aged 35 to 49, sexual partners (vs. children and other household members), and female contacts and differences in test positivity by region of the country 27. These results suggest that local differences in HIV prevalence and access to HTS or differences in testing behaviours or HIV incidence by gender and age may be useful for prioritizing limited resources for PNS.

## 3 Client, community and provider perspectives on PNS

Understanding the perspectives of communities affected by HIV, potential clients and their partners, and healthcare workers who are or will be performing PNS is necessary for the development and implementation of effective, safe and scalable programmes. Articles from this supplement provide new perspectives on PNS for key and vulnerable populations 24 and pregnant and postpartum women 31 as well as how PNS can be integrated into both HIV care and prevention programmes 32, 33.

Global leaders and advocates from diverse communities affected by HIV provide practical guidance on how to engage their communities and maintain a focus on human rights to deliver ethical and effective PNS, particularly for key populations 24. Odoyo et al. 32 discuss perspectives and experiences from HIV care providers in Kenya, who found that PNS and PrEP have the potential to be synergistic in the HIV care setting. PNS was seen as an opportunity to increase uptake of couple‐based prevention, including offering PrEP to negative partners and retaining partners with HIV in ART services. Availability of PrEP within HIV treatment services also increased the motivation of providers to offer PNS and of clients with HIV to accept PNS and share their status with their partners. Also from the Kenyan HIV care setting, Monroe‐Wise et al. 33 found that barriers to PNS among people with established HIV infection and on treatment revolved primarily around fear of repercussions stemming from sharing HIV diagnoses with their partners, particularly when the client had not shared their status with the partner immediately after diagnosis. Providers and clients were able to identify multiple strategies for addressing these concerns and employ PNS safely and effectively. Finally, pregnant women, their male partners, healthcare workers and policymakers from Zambia and Malawi considered multiple options for testing male partners of pregnant women 31. As no single approach was overwhelmingly preferred within or across stakeholder groups and all had multiple pros and cons, the authors concluded that a choice‐based approach may result in greatest uptake of these services and enhance their impact. Taken together, these perspectives can support the development of new strategies and best practices to ensure the safe and effective delivery of PNS to all people with HIV on an ongoing basis as recommended by WHO and PEPFAR guidelines.

## 4 PNS for key populations

To date, most of the evidence for the effectiveness and safety of PNS in LMICs has come from programmes and studies in the general population. There are concerns that this evidence may not be generalizable to key populations in rights‐constrained settings where criminalization and stigma are prevalent, confidentiality is frequently breached, and there is limited access to rights‐based and culturally sensitive services. Taken together, these concerns may affect the acceptability, effectiveness and potential social harms associated with assisted and passive partner notification strategies. Additionally, differences in partnership types (e.g. transactional partnerships or drug‐sharing contacts) and community norms across populations may further influence the acceptability and delivery of PNS. Formative evidence indicates that PNS may be acceptable to some members of key populations in LMIC 16, but may be challenging in others 34, 35. Encouragingly, PNS approaches have also been successfully provided to gay, bisexual, and other men who have sex with men (MSM) and people who inject drugs (PWID) in several high‐income countries for many years and lessons learned from these programmes may inform scale‐up in LMICs.

This supplement includes three articles describing the implementation of PNS programmes serving key populations. From Vietnam, Nguyen et al. 29 describe assisted PNS within a community‐led, peer educator‐delivered HTS programme that serves MSM, PWID, female sex workers and transgender people. Uptake of PNS was high (81%) and the HIV positivity rate among contacts was 42%, greater than the rate from HTS delivered by lay providers and HIV self‐testing. From Pakistan, Malik et al. 26 report on an assisted partner notification strategy for reaching spouses of PWID with ongoing HIV testing, prevention and care services through field outreach and sensitization of heads of family (often mothers‐in‐law) to overcome barriers related to local culture and family structures. This programme reached the spouses of 71% of married index clients but had relatively low positivity (9%). Finally, Little et al. 27 describe the integration of assisted PNS into three types of activities within PWID‐focused programmes in Kazakhstan, the Kyrgyz Republic and Tajikistan. These activities include: (1) HTS for people newly diagnosed with HIV; (2) tracing of individuals with HIV who were lost‐to‐follow‐up in order to re‐engage them in HIV care and (3) HIV care for individuals with HIV who are receiving HIV care services but are not yet taking ART. Compared to a period with passive referral only, implementation of assisted PNS increased the number of partners tested and newly diagnosed with HIV per index client, significantly improving the efficiency of HTS. Overall, these evaluations provide additional evidence of the acceptability and potential effectiveness of PNS for key populations. They also provide examples of how PNS can be tailored to the local context and populations being served. The Vietnam programme, in particular, highlights the role that engaging affected communities can have in the development and implementation of successful PNS programmes, a message consistent with one of the key recommendations from the viewpoint by members of affected communities 24.

## 5 Social harms following PNS

A key concern raised by advocates and providers alike about PNS implementation and scale‐up, particularly around the provider referral method, is the potential for social harms, such as IPV, relationship dissolution and breaches of confidentiality. New results from the Cameroon Baptist Convention Health Services’ PNS programme 28, 36 add to previous evidence from research studies and programme evaluations 11, 12, 13, 14, 37, 38 that facility‐based assisted PNS for the general population result in few adverse events, even in settings with high background rates of gender‐based violence. However, as described in the previous section and in‐depth in the community viewpoint 24, it has not been clear to what extent these findings can be applied to PNS programmes serving key populations in rights‐constrained settings, where the consequences of PNS may be complicated by structural and sociocultural factors affecting the lives and partnerships of these groups. Encouragingly, in this supplement, an evaluation of a community‐led HTS programme with integrated assisted PNS for key populations in Vietnam – primarily MSM and PWID – found no evidence of social harms related to partner notification 29.

To mitigate the risk of social harms, it will be critical for PNS programmes to continue to screen for IPV prior to offering PNS to index clients. Options for clients who report IPV in the current relationship include partner notification (which does not require disclosure to the partner and can be conducted anonymously) or a delay in participating in PNS until the safety of the index client can be assured. In addition, all index clients who report IPV should be actively linked to gender‐based violence services to get the care and support they need. HIV testing programmes should also monitor social harms resulting from participation in PNS to ensure that protections have been put in place to protect the safety of index clients and their partner(s). Additional research is needed to ensure the safety of PNS among key populations in different programme models and settings. Articles by Ayala et al. 24 and Wamuti et al. 36 in this supplement present direct, practical recommendations for ensuring safe, respectful and effective delivery of PNS.

## 6 Training is critical

Effective, culturally competent training for health workers and lay providers is critical to the successful and ethical implementation of PNS programmes. As outlined by Han et al. 39 in this supplement, successful training programmes for PNS typically incorporate a combination of didactic and active skills‐based learning with on‐the‐job mentorship, monitoring and evaluation and refresher trainings. This article also presents common topics of PNS training, from the rationale and principles of PNS to counselling and communication skills to ethical issues, as well as strategies for implementing and sustaining high‐quality training programmes. Equally important are the selection criteria used to determine which healthcare workers should provide PNS. Ideally, PNS providers should be experienced counsellors with at least one year of experience conducting HTS and should have the psychologic composure to offer PNS in a tactful, patient and open‐minded manner. Supportive supervision and mentoring for newly trained PNS providers are also key for sharing best practices and avoiding burn‐out.

Tembo et al. 40 demonstrate the potential effectiveness of a one‐day behavioural skills‐building training for improving the delivery of a client referral model for partner testing in Malawi. Although WHO recommends assisted PNS, many index clients select client referral as their preferred strategy for notifying partner(s), and some programmes continue to rely exclusively or primarily on client referral strategies due to national policies, lack of programme capacity to offer assisted referral, and other concerns. Strategies for improving the effectiveness of passive referral are therefore needed. In a pre/post evaluation, Tembo and colleagues found that a one‐day training to improve health worker skills in offering and supporting clients in delivering family referral slips resulted in significant increases in the recorded number of contacts that were elicited, received HIV testing, and were diagnosed as HIV positive. This study suggests that a brief skills‐based training has the potential to increase the effectiveness of passive referral, although further study will be necessary to assess its potential value within PNS programmes offering assisted referral.

## 7 Conclusions

Everyone with HIV benefits from early diagnosis and linkage to ART for their own health and to prevent transmission to uninfected partners and infants. As countries reach the UNAIDS 90‐90‐90 targets – the first being 90% of people with HIV knowing their status and the second linking to and taking ART 22 – there remain significant gaps, and new approaches are needed to address these gaps. Men and key populations, in particular, continue to have less access to HIV testing and treatment services. Articles in this supplement contribute to the growing evidence demonstrating that PNS can be a successful strategy in reaching undiagnosed populations more efficiently and effectively.

As with all testing approaches, PNS must be delivered in a way that avoids social harms. Proper training of providers on how to safely provide PNS and support for the index clients who receive PNS services are therefore critical. For example, how do we do provide PNS safely to index clients in abusive relationships? Articles in this supplement have provided some best practices for mitigating the risk of social harms. Programmes should also consider using WHO guidance on assessing IPV risk 41 and continue to monitor the experience of social harms following PNS to ensure that these services are offered in ways that respects the human rights of index clients and their partner(s) and preserves their safety.

Huge advances in HIV prevention, testing and treatment have been made in the 35 years since the HIV epidemic began. HIV should now be considered a manageable chronic condition, and HIV transmission should be easily preventable by supporting people with HIV to achieve viral suppression and engaging people at risk in the increasing options for HIV prevention. However, HIV epidemic control cannot be achieved unless people with HIV are diagnosed early, rapidly linked to HIV treatment services and have HIV prevention services readily availability for their HIV‐negative at‐risk partners. By improving HIV testing coverage among underserved populations and those most at risk for HIV infection, PNS can play a key role in achieving these objectives and, ultimately, HIV epidemic control.

## Competing interests

The authors have no competing interests to declare.

## Authors’ contributions

DK, VW and RB drafted the initial manuscript. All authors critically reviewed the manuscript, suggested revisions and editorial changes, and approved the final version.

8

**A note on terminology**

*PNS*, or contact tracing, is defined as a voluntary process whereby a trained provider asks people diagnosed with HIV often known as the “index case” about their sexual partners and/or drug injecting partners and then, if the HIV‐positive client agrees, offers these partners HTS. Partner notification is provided using passive or assisted approaches.

*Passive HIV PNS* or “client referral” refer to when HIV‐positive clients are encouraged by a trained provider to disclose their HIV status to their sexual and/or drug injecting partners by themselves, and to encourage their partner(s) to be tested for HIV given their potential exposure to HIV infection.

*Assisted HIV PNS* refer to when consenting HIV‐positive clients are assisted by a trained provider to disclose their status or to anonymously notify their sexual and/or drug injecting partner(s) of their potential exposure to HIV infection. The provider then offers HIV testing to these partner(s). *Assisted partner notification is done using contract referral, provider referral or dual referral approaches*.

- *Contract referral*: HIV‐positive clients enter into a contract with a trained provider and agree to disclose their status and the potential HIV exposure to their partner(s) by themselves and to refer their partner(s) to HTS within a specific time period. If the partner(s) of the HIV‐positive individual does not access HTS or contact the health provider within that period, then the provider will contact the partner(s) directly and offer voluntary HTS.
- *Provider referral*: With the consent of the HIV‐positive client, a trained provider confidentially contacts the person's partner(s) directly and offers the partner(s) voluntary HTS.
- *Dual referral*: A trained provider accompanies and provides support to HIV‐positive clients when they disclose their HIV status to their partner(s). The provider also offers voluntary HTS to the partner(s).

*Index testing* usually refers more broadly to offering HIV testing to all contacts of an index client including sexual and needle and syringe‐sharing partners, biological children, and, in the case of an HIV‐positive child, the mother and biologic siblings.

*Social network testing* is where an HIV‐positive client is asked to enlist people in their social networks who may benefit from HIV testing to participate in HIV testing services 42, 43. These contacts can, in turn, recruit their social contacts who are also at risk for HIV.
